# Steroids and Malignancy Increase Local Heparanase and Decrease Markers of Osteoblast Activity in Bone Tissue Microcirculation

**DOI:** 10.3390/biom14121506

**Published:** 2024-11-26

**Authors:** Keren Asayag, Eli Peled, Mai Assalia, Yonatan Crispel, Chen Yanovich, Haim Cohen, Anat Keren-Politansky, Yona Nadir

**Affiliations:** 1 Thrombosis and Hemostasis Unit, Rambam Health Care Campus, Haifa 3109601, Israel; kerenasayag0@gmail.com (K.A.); mai.z.assalia@gmail.com (M.A.); y_crispel@rambam.health.gov.il (Y.C.); hionobich@gmail.com (C.Y.); haimge1@walla.co.il (H.C.); anat_keren@rambam.health.gov.il (A.K.-P.); 2 The Ruth and Bruce Rappaport Faculty of Medicine, Israel Institute of Technology—Technion, Haifa 3109601, Israel; e_peled@rambam.health.gov.il; 3 Orthopedic Division, Rambam Health Care Campus, Haifa 3109601, Israel

**Keywords:** bone, microcirculation, thrombosis, heparanase, steroids, malignancy

## Abstract

Bone metastasis and steroids are known to activate the coagulation system and induce osteoporosis, pathological bone fractures, and bone pain. Heparanase is a protein known to enhance the hemostatic system and to promote angiogenesis, metastasis, and inflammation. The objective of the present study was to evaluate the effects of steroids and malignancy on the coagulation factors and osteoblast activity in the bone tissue. The effects of dexacort and malignant medium were evaluated in osteoblasts derived from human bone marrow mesenchymal stem cells and human umbilical vein endothelial cells (HUVECs). The bones of mice treated with dexacort for 1 month were studied. Bone biopsies of ten patients with bone metastasis, ten with steroid-induced avascular necrosis (AVN), and ten with osteoarthritis were compared to ten controls. We found that dexacort and malignant medium significantly increased the heparanase levels in osteoblasts and HUVECs and decreased the levels of alkaline phosphatase (ALKP). Peptide 16AC, derived from heparanase, which interacts with tissue factor (TF), further increased the effect, while peptide 6, which inhibits interactions between heparanase and TF, reversed the effect in these cells. The bone microcirculation of mice treated with dexacort exhibited significantly higher levels of heparanase, TF, TF pathway inhibitor (TFPI), TFPI-2, thrombin, and syndecan-1, but reduced levels of osteocalcin and ALKP. The pathological human bone biopsies’ microcirculation exhibited significantly dilated blood vessels and higher levels of heparanase, TF, TFPI, TFPI-2, and fibrin. In summary, steroids and malignancy increased the activation of the coagulation system in the bone microcirculation and reduced the osteoblast activity. Heparanase inhibitors should be further investigated to attenuate bone fractures and pain.

## 1. Introduction

The blood supply of the bone and bone marrow is interconnected through a network of vessels. Arteries penetrating the bone flow into the marrow cavity, while blood from marrow sinuses and veins leaves the tissue via the bone. The blood eventually drains mainly through one or more nutrient veins [1,2,3]. Thus, the bone and bone marrow may be considered one organ. Bone metastasis and steroids are known to induce osteoporosis, pathological bone fractures, and bone pain [4,5]. These two etiologies are also known to activate the coagulation system and promote thrombosis [6].

Heparanase, a β-D-endoglucuronidase enzyme found to cleave the heparan sulfate side chains on the cell surface and in the extracellular matrix [7,8,9], was later shown to be an enhancer of inflammation, angiogenesis, and metastasis [10,11,12,13]. We previously demonstrated that heparanase might also affect the hemostatic system in a non-enzymatic manner [14,15]. Recently, several groups supported the involvement of heparanase in coagulation system activation [16,17,18,19,20,21,22]. Our findings demonstrated that heparanase directly enhances tissue factor (TF) activity, leading to increased factor Xa production and subsequent activation of the coagulation system [15]. Our earlier studies showed that heparanase up-regulated the expression of TF [14] and interacted with the tissue factor pathway inhibitor (TFPI) on the cell surface membrane of endothelial and tumor cells, resulting in TFPI dissociation and increased cell surface coagulation activity [23]. We recently detected the procoagulant domain in the heparanase protein that interacts with TF and generated peptides derived from that site (peptides 16 and 16AC). Additionally, we revealed the domain in TFPI-2 that is capable of inhibiting the interaction between heparanase and the TF complex, and we generated a peptide derived from that site (peptide 6) [24,25]. Interestingly, the inhibition of the latter complex induces reduced tumor growth and vascularization [26], implying that the interaction between heparanase and TF also exerts non-hemostatic effects. In the present study, we investigated the effect of steroids and malignancy conditions on the coagulation factors and osteoblast activity in the bone tissue.

## 2. Materials and Methods

### 2.1. Reagents and Antibodies

A single-chain GS3 heparanase gene construct, comprising the 8 and 50 kDa heparanase subunits (8 + 50), was purified from the conditioned medium of baculovirus-infected cells. GS3 heparanase assayed for the presence of bacterial endotoxin (Biological Industries, Beit Haemek, Israel) using the gel-clot technique (limulus amebocyte lysate–LAL test) was found to contain <10 pg/mL of endotoxin [14]. Antibody 733 was raised in rabbits against a 15 amino acid peptide that maps at the N-terminus of the 50 kDa heparanase subunit [27]. Martin Scarlet Blue (MSB) staining was purchased from Cancer Diagnostics (Durham, UK). The amino acid sequences of the peptides: amid-GSKRRKLRVYLHCT-acetyl (peptide 16AC). Amid and acetyl residues prevent peptide degradation and enhance activity. Peptide 16AC derives from the procoagulant domain of heparanase. Peptide AEICLLPLDYGPCR (peptide 6), which derives from TFPI-2 and inhibits the interaction between TF and heparanase.

### 2.2. Study Group

This study was approved by the Institutional Review Board of the Rambam Health Care Campus (no. 0304-18-RMB). Samples were obtained from the pathology department and details from the medical charts. Written informed consent was not required. The bone biopsies of 40 patients were studied: ten cases of bone metastasis of carcinoma, ten cases of avascular necrosis (AVN) of the femur head due to steroids, and ten cases of osteoarthritis of the femur head. The biopsies were taken at the beginning of femur surgery. Ten cases of diffuse large cell lymphoma without bone or bone marrow involvement constituted the control group, which were harvested during the bone marrow (BM) biopsy procedure during staging. Inclusion criteria: age > 18, with use of anti-aggregate therapy permitted. Exclusion criteria: pregnancy, hormonal therapy, and use of anticoagulation therapy (prophylactic or therapeutic dose). 

### 2.3. Mouse Model

This study was approved by the Technion Ethics Committee for Animal Research (approval code IL0510319) and the procedures followed were in accordance with institutional guidelines. Institute of Cancer Research (ICR) mice with no specific genetic background were used in this study. All the experiments were performed in seven- to eight-week-old male mice in order to avoid a hormonal effect on the coagulation factors.

### 2.4. MSCs Isolation and Osteoblasts Differentiation

Human BM pellet (2 mL) from the above-mentioned control group was added to 20 mL MSC culture medium and incubated at 37 °C, 5% CO_2_ overnight. The next day, the cells were washed twice with phosphate-buffered saline (PBS) and grown in mesenchymal stem cell (MSC) medium. The MSCs were further differentiated to osteoblasts with osteogenic medium. After 3 weeks, the osteoblasts were evaluated by specific staining.

### 2.5. Alkaline Phosphatase (ALKP) Staining for Detection of Osteoblasts

The cells were washed with PBS and then fixed using neutral buffered formalin (10%). After 1 min, the cells were washed with washing buffer (0.05% Tween with PBS). The cells were stained using 10 mL 5-bromo-4-chloro-3-indolyl phosphate (BCIP)/nitro blue tetrazolium (NBT) substrate solution (SigmaFast^TM^ BCIP-NBT; Sigma Aldrich, Rehovot, Israel) and incubated at room temperature in the dark. After 10 min, the cells were washed with washing buffer, followed by washing with PBS. The staining results were evaluated.

### 2.6. Cell Cultures

Human umbilical vein endothelial cells (HUVECs) were grown in M-199 medium Earle’s salts (Biological Industries, Beit Haemek, Israel) and supplemented with 20% fetal bovine serum (FBS), 1% L-glutamine and 1% penicillin, streptomycin, and amphotericin, in addition to endothelial growth factor 100 µg/mL (Biomedical Tech, Zotal, Israel). Human mesenchymal stem cells (MSCs) and osteoblast cells were maintained in MEM-alpha (Biological Industries, Beit Haemek, Israel) and supplemented with 10% FBS, 1% L-glutamine, and 1% penicillin, streptomycin, and amphotericin. Osteoblast differentiation from the MSCs was induced by MSCs medium enriched with 1% β-glycerolphosphate, 1% ascorbic acid, and 0.0002% dexamethasone (Sigma Aldrich, Israel) for at least 3 weeks. All the cells were cultured at 37 °C, 5% CO_2_. MCF-7 human breast carcinoma cells, MDA-231 human breast carcinoma, and A549 human lung adenocarcinoma were purchased from the American Type Culture Collection (ATCC, Manassas, VA, USA). The cells were grown in Dulbecco’s modified Eagle’s medium (Biological Industries, Beit Haemek, Israel), supplemented with 10% fetal calf serum and antibiotics.

### 2.7. Cell Lysis

A 1 mL culture dish was placed on ice. The cells were washed with ice-cold PBS, followed by an addition of 0.5 mL ice-cold lysis buffer (50 mM Tris-HCl, 150 mM NaCl, and 0.5% Triton X-100, at pH 7.4) with protease inhibitor (1:1000) for 10 min. The cell suspension was centrifuged in a microcentrifuge at 15,000× *g* for 10 min, 4 °C. The supernatant was aspirated and placed in a fresh tube kept on ice, and the pellet was discarded.

### 2.8. Immunostaining

The cells were seeded on slides in 150 mm plates. The next day, the cells were fixed using formaldehyde 4% for 20 min, followed by washing with PBS twice. The blocking steps included successive incubation in 3% H_2_O_2_ in methanol for 20 min, washing with PBS three times for 3 min each, and incubation with 10% normal goat serum (NGS) in PBS for 30 min to block non-specific binding. The cells were incubated (90 min, 25 °C) with anti-heparanase polyclonal antibody (733), diluted 1:250 in blocking solution. Antibody 733 originates from the C-terminus of the 50 kDa human heparanase subunit; it preferentially recognizes the 50 kDa heparanase subunit versus the 65 kDa latent pro-enzyme. Other antibodies that were used for staining: anti-TF polyclonal antibody (1:100, Santa Cruz, CA, USA), anti-TFPI-1 polyclonal antibody (1:100, Santa Cruz, CA, USA), or anti-TFPI- 2 polyclonal antibody (1:100, Santa Cruz, CA, USA), anti-thrombin polyclonal antibody (1:50, Santa Cruz, CA, USA), osteocalcin polyclonal antibody (1:50, Santa Cruz, CA, USA), and syndecan-1 polyclonal antibody (1:50, Santa Cruz, CA, USA). The slides were extensively washed with PBS and incubated with a secondary reagent (Envision kit, Dako, Glostrup, Denmark) according to the manufacturer’s instructions. Following additional washes, the color was developed with the AEC reagent (Dako, Glostrup, Denmark) and sections were counterstained with hematoxylin and mounted.

Analyses of the immunostaining results were performed by two of the authors, who were unaware of the slide allocation. Discrepancies in the analyses were reconciled following the assessment by a third reviewer. Five high-power fields were evaluated in each stained slide. The staining intensity was scored as follows: 0, no staining; 1, weak intensity; 2, moderate intensity; and 3, marked intensity.

### 2.9. Heparanase Enzyme-Linked Immunosorbent Assay (ELISA)

The wells of microtiter plates were coated (18 h, 4 °C) with 2 μg/mL of anti-heparanase monoclonal antibody 4B11 in 50 μL coating buffer (0.05 M Na_2_CO_3_, 0.05 M NaHCO_3_, pH 9.6). The plate was covered with adhesive plastic and incubated overnight at 4 °C. The next day, the wells were blocked with 2% BSA in PBS for 1 h at room temperature (23 °C). Diluted samples (100 μL) were loaded in duplicates and incubated for 2 h at room temperature, followed by the addition of 100 μL polyclonal antibody 63 IgG (1 μL/mL) for an additional period of 2 h at room temperature. HRP-conjugated goat anti-rabbit IgG (1:20,000) in blocking buffer was added (1 h, room temperature) and the reaction was visualized by the addition of 100 μL chromogenic substrate (TMB; MP Biomedicals, Eschwege, Germany) for 15 min. The reaction was stopped with 100 μL H_2_SO_4_ and the absorbance at 450 nm was measured using an ELISA plate reader (Power Wave XS, BIO-TEK, Winooski, VT, USA). The plates were washed four times with washing buffer (PBS, pH 7.4 containing 0.1% (*v/v*) Tween 20) after each step. As a reference for quantification, a standard curve was established by serial dilutions of recombinant 8 + 50 GS3 heparanase (390 pg/mL–25 ng/mL) [28].

### 2.10. Statistical Analysis

The data were evaluated using the SPSS software for Windows, version 13.0 (SPSS Inc., Chicago, IL, USA). Statistics were calculated by the Mann–Whitney U test. The values were reported as the median and range. The significance level was set at *p* < 0.05.

## 3. Results

### 3.1. Dexacort or Medium of Malignant Cells Increased Heparanase Expression and Reduced ALKP in Osteoblast Cells

**Figure 1:**
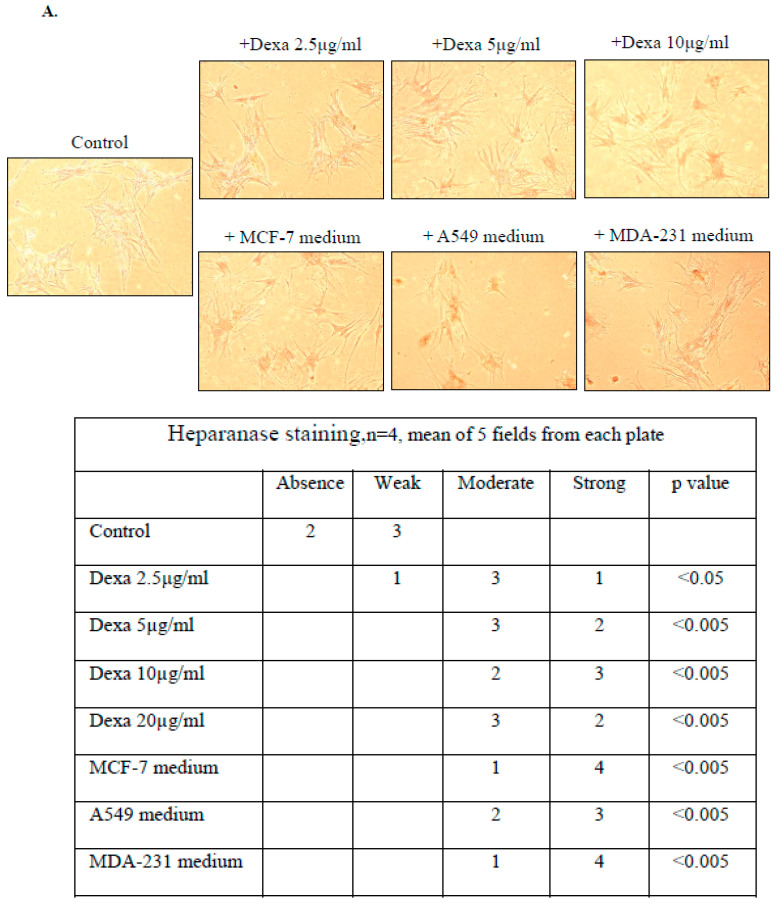

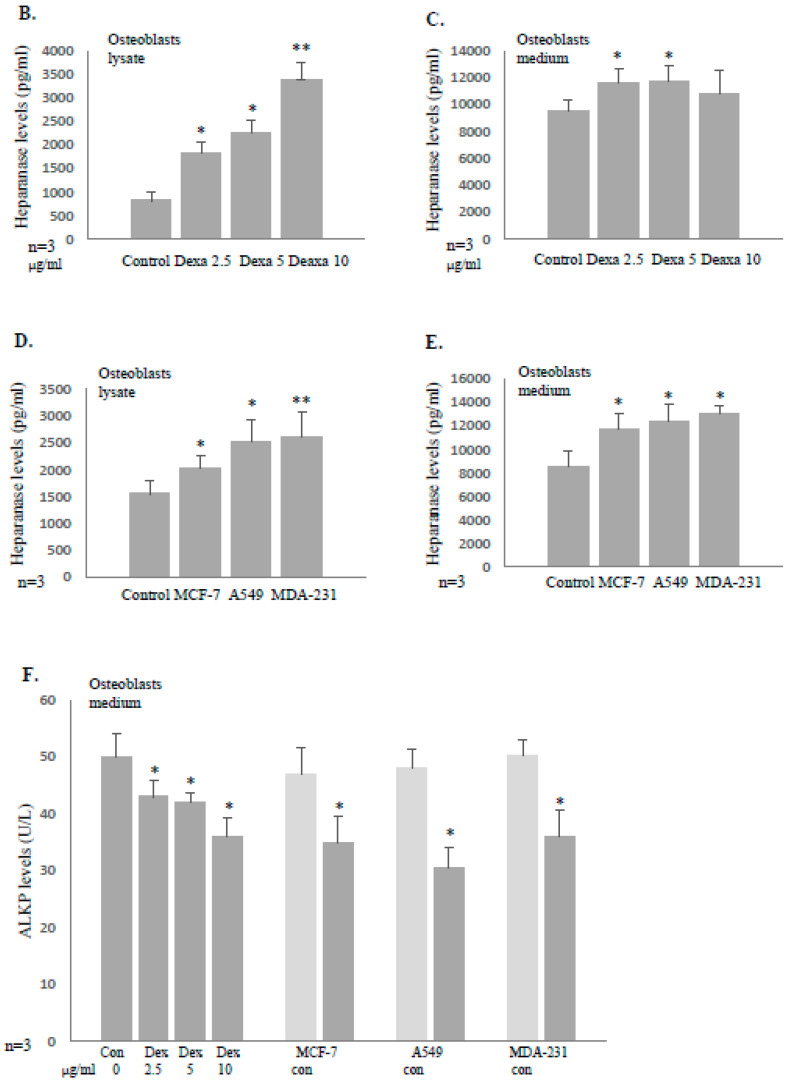
Dexacort or medium of malignant cells increases heparanase expression and reduces ALKP in osteoblast cells. Human MSCs were isolated from human BM pellet and differentiated to osteoblasts with osteogenic medium, as described in the Methods section. After 3 weeks, the osteoblasts were incubated for 48 h under the following conditions: 1. control (1000 μL osteogenic medium); 2. dexacort in increasing concentrations equivalent to therapeutic doses (2.5 µg/mL, 5 µg/mL, 10 µg/mL) and osteogenic medium; and 3. osteogenic medium (1000 µL) derived from overnight incubation with three malignant cell lines (MCF-7—human breast carcinoma, A549—human lung adenocarcinoma, and MDA-231—human breast carcinoma). (**A**) Immunohistochemistry staining revealed that osteoblasts incubated with dexacort or medium from malignant cells showed a significantly higher level of heparanase. The contiguous table shows the indicated protein staining intensity in the cells. Significance was determined by the Mann–Whitney U test. Representative images were visualized through ×10 magnitude, with 0.82 MDC objective lens, captured with a Nikon E995 digital camera (Nikon, Tokyo, Japan), and processed with Adobe Photoshop software (Adobe Systems, San Jose, CA, USA). The heparanase levels in the mediums and lysates were tested by ELISA assay, as described in the Methods. The results show increased heparanase levels in both the osteoblast lysates and medium after incubation with dexacort (**B**,**C**) or medium from malignant cells (**D**,**E**). ALKP levels by spectrophotometry analysis that indicate the osteoblast activity showed that dexacort and malignant medium reduced the levels of ALKP significantly (**F**). The effect of malignant medium was compared to the level of ALKP in the medium before incubation with osteoblasts (light gray). Significance was determined by the Mann–Whitney U test. All the assays were performed in triplicate. Results represent median and range of three experiments. * *p* < 0.05, ** *p* < 0.005.

Steroid drugs as well as malignant cells are known to cause damage to bone tissue strength and induce osteoporosis. In order to investigate the effect of these two factors on heparanase, dexacort or medium of malignant cells were incubated with osteoblast cells for 48 h. Immunohistochemistry staining (Figure 1A) and ELISA assay (Figure 1B–E) revealed increased heparanase levels in osteoblast cells incubated with dexacort or medium of malignant cells (Figure 1). In parallel, the ALKP levels, which indicate osteoblast activity, significantly decreased under the condition of dexacort or malignant medium (Figure 1F).

### 3.2. Heparanase Expression Was Increased and ALKP Levels Decreased in Osteoblast Cells When Incubated with the Heparanase Procoagulant Peptide 16AC

**Figure 2:**
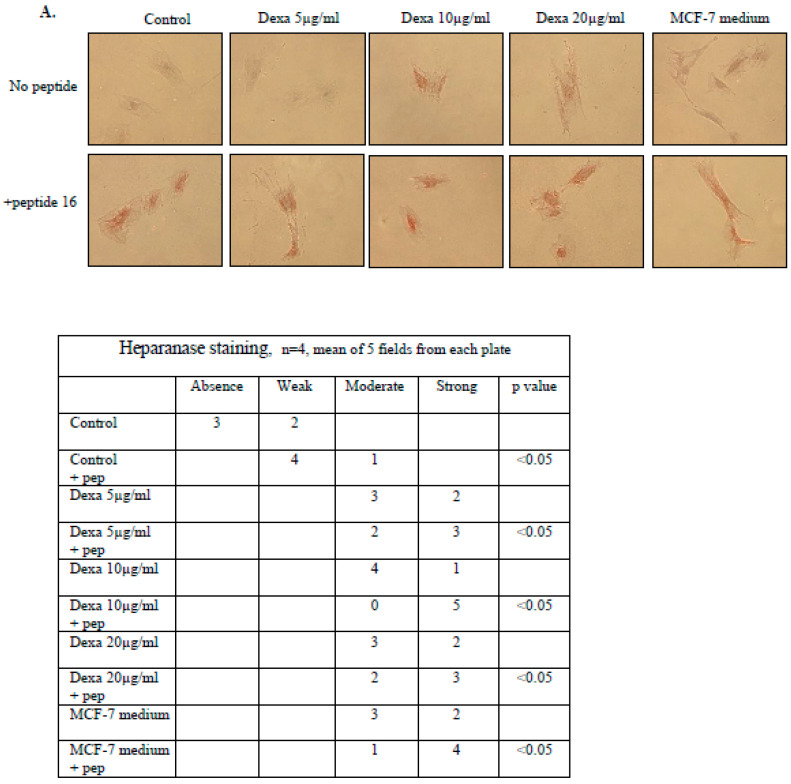

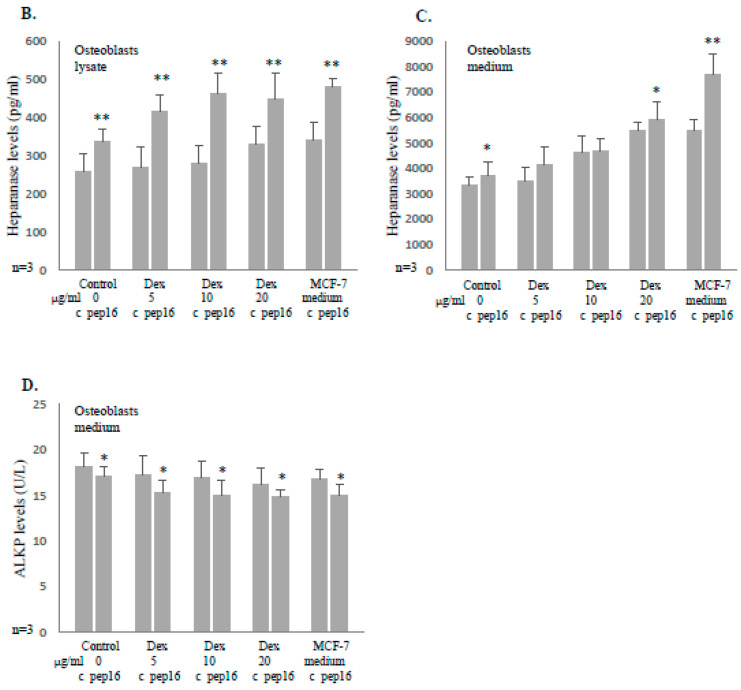
Heparanase expression was increased and ALKP decreased in osteoblast cells when incubated with the heparanase procoagulant peptide 16AC. MSCs were isolated from human BM pellet and differentiated to osteoblasts with osteogenic medium. After 3 weeks, the osteoblasts were incubated for 48 h under the following conditions: 1. control (1000 μL osteogenic medium); 2. dexacort in increasing concentrations equivalent to therapeutic doses (5 µg/mL, 10 µg/mL, and 20 µg/mL) in osteogenic medium; and 3. osteogenic medium (1000 µL) derived from overnight incubation with malignant cell line (MCF-7). Peptide 16AC (designated 16, 5 μg/mL) was added to half of the plates. (**A**) Immunohistochemistry staining. The results revealed a significant increase in heparanase expression in osteoblast cells incubated with peptide 16AC and dexacort or medium from malignant cells. The contiguous table shows the indicated protein staining intensity in the cells. Significance was determined by the Mann–Whitney U test. Representative images were visualized through ×50 magnitude, with 0.82 MDC objective lens, captured with a Nikon E995 digital camera (Nikon, Tokyo, Japan), and processed with Adobe Photoshop software (Adobe Systems, San Jose, CA, USA). (**B**,**C**) The heparanase levels in mediums and lysates were tested by ELISA assay. The results show that peptide 16AC has an additive effect on the heparanase levels in osteoblast cell lysate and medium incubated with dexacort or malignant cells medium. All the assays were performed in triplicate. The results represent the median and range of three experiments. (**D**) The ALKP level was measured in the medium of the above-described experiment. The results indicate that peptide 16AC has an additive effect on decreasing the ALKP levels in osteoblast cells incubated with dexacort or malignant cells medium. The results represent the median and range of three experiments. * *p* < 0.05, ** *p* < 0.005.

After we demonstrated that dexacort or malignant medium increased heparanase expression (Figure 1), we explored the impact of the procoagulant peptide 16AC. This peptide is derived from the heparanase protein and is the site of interaction between TF and heparanase. We recently showed that this interaction not only enhances activation of the coagulation system but also induces intracellular non-hemostatic effects [26]. Peptide 16AC was added to osteoblast cells incubated with dexacort or malignant medium. Immunohistochemistry staining (Figure 2A) and ELISA assay (Figure 2B,C) revealed that peptide 16AC has an additive effect on heparanase expression in osteoblast cells incubated under these conditions. In parallel, the results showed that peptide 16AC has an additive effect on decreasing the ALKP levels in osteoblast cells incubated with dexacort or malignant cells medium (Figure 2D). The results indicate that multiple triggers can further increase the heparanase level in osteoblasts and decrease the osteoblast activity, possibly by a number of pathways.

### 3.3. Heparanase Inhibitory Peptide 6 Attenuates the Increase of Heparanase and Reduction of ALKP Induced by Dexacort and Medium from Malignant Cells in Osteoblast Cells

**Figure 3:**
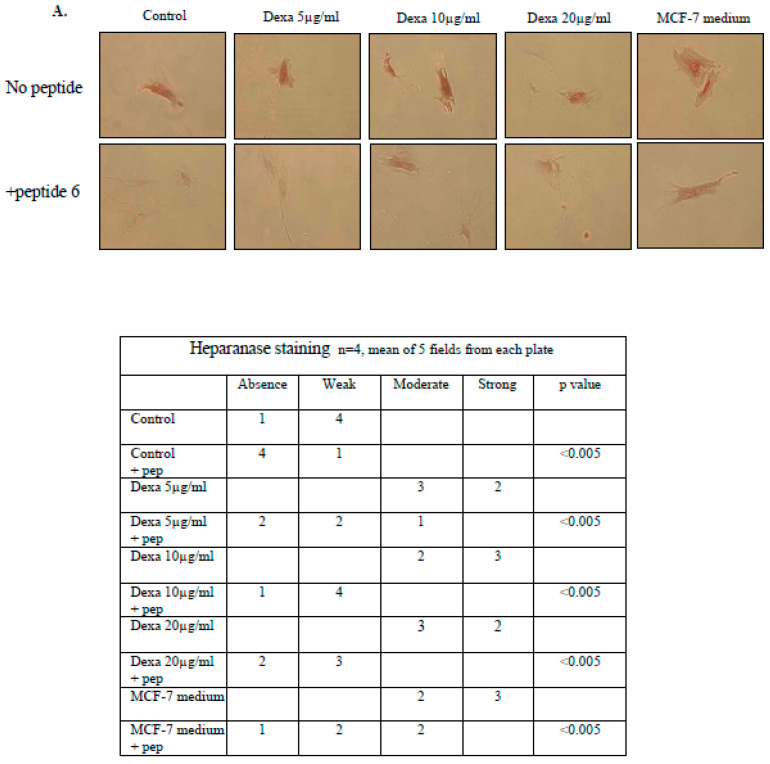

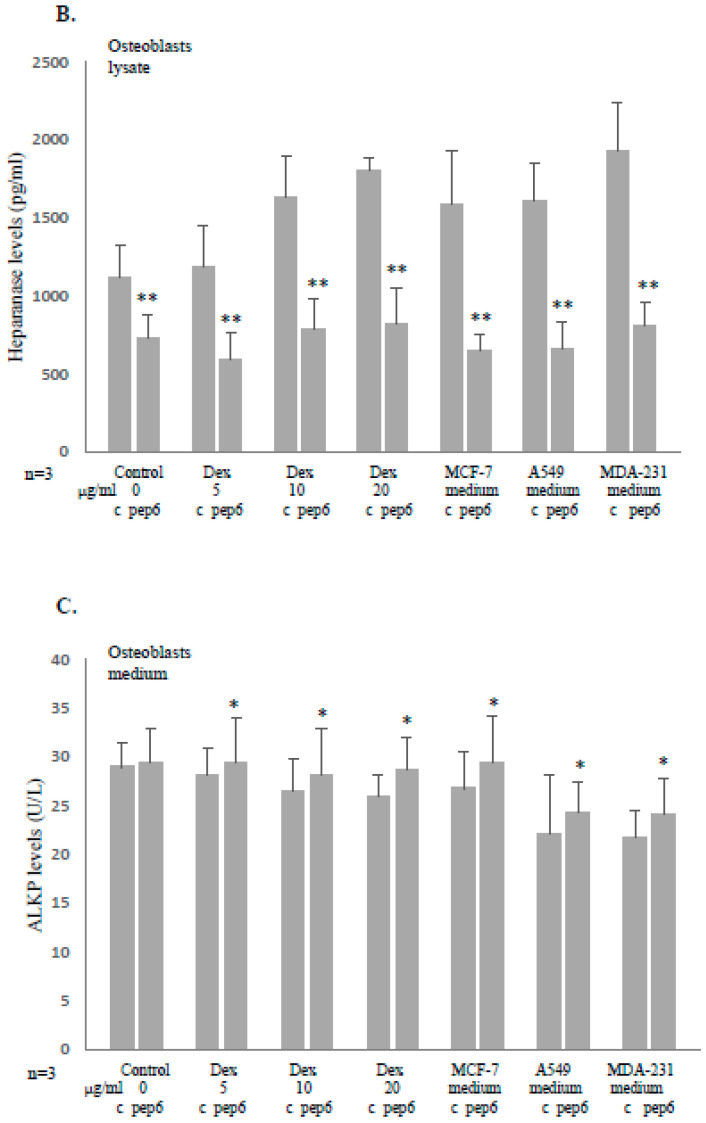
Heparanase inhibitory peptide 6 attenuates the increase in heparanase and reduction in ALKP induced by dexacort and medium from malignant cells in osteoblast cells. MSCs were isolated from human BM pellet and differentiated to osteoblasts with osteogenic medium. After 3 weeks, osteoblasts were incubated for 48 h under the following conditions: 1. control (1000 μL osteogenic medium); 2. dexacort in increasing concentrations equivalent to therapeutic doses (5 µg/mL, 10 µg/mL, and 20 µg/mL) and osteogenic medium; and 3. osteogenic medium (1000 µL) was derived from overnight incubation with malignant cells (**A**,**B**). Peptide 6 (50 μg/mL) was added to half of the plates. (**A**,**B**) Immunohistochemistry staining and ELISA. The results revealed that peptide 6 attenuated the heparanase increase induced by dexacort and malignant medium in osteoblast cells. Contiguous table shows the indicated protein staining intensity in the cells. Significance was determined by the Mann–Whitney U test. Representative images were visualized through ×50 magnitude, with 0.82 MDC objective lens, captured with a Nikon E995 digital camera (Nikon, Tokyo, Japan), and processed with Adobe Photoshop software (Adobe Systems, San Jose, CA, USA). (**C**) ALKP level. The results indicate that peptide 6 reduced the ALKP level when added to osteoblast cells incubated with dexacort or medium from malignant cells. The results represent the median and range of three experiments.* *p* < 0.05, ** *p* < 0.005.

The inhibitory peptide 6 was shown to inhibit the interaction between TF and heparanase. We recently demonstrated that not only does peptide 6 attenuate activation of the coagulation system but also reduces intracellular non-hemostatic effects induced by the TF–heparanase complex [26]. In the present study, peptide 6 was added to osteoblast cells incubated with dexacort or malignant medium. Immunohistochemistry staining (Figure 3A) and ELISA assay (Figure 3B) revealed that peptide 6 reduced the increase in heparanase induced by dexacort and malignant medium in osteoblast cells. In parallel, it significantly increased the osteoblast activity, as indicated by higher levels of ALKP (Figure 3C). The results indicate that TF–heparanase complex inhibition is a major mechanism involved and may potentially be investigated as a protector of bone tissue.

### 3.4. Peptide 6 Reduces Heparanase Level and Increases ALKP Level Induced by Dexacort in HUVECs

**Figure 4:**
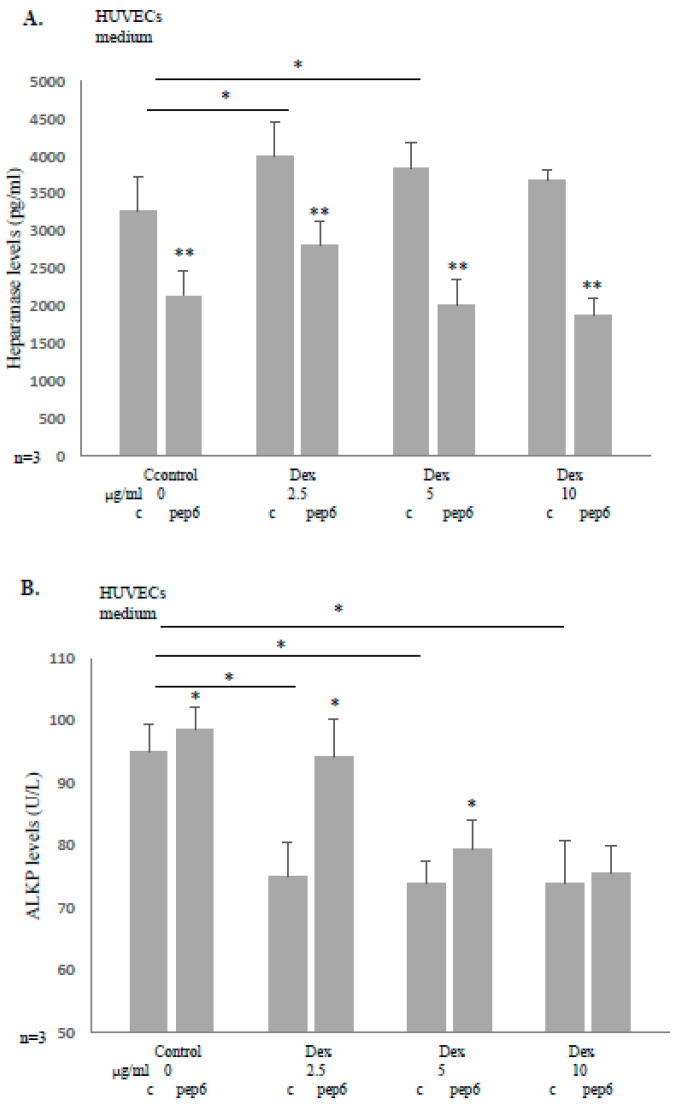
Peptide 6 reduces heparanase level and increases ALKP level induced by dexacort in HUVECs. (**A**) HUVECs at the 5th passage were incubated for 48 h under the following conditions: 1. control (1000 μL M-199 medium); and 2. dexacort in increasing concentrations equivalent to therapeutic doses (2.5 µg/mL, 5 µg/mL, and 10 µg/mL) and M-199 medium. Heparanase inhibitory peptide 6 (50 μg/mL) was added to half of the plates. The results show that dexacort increased and peptide 6 reduced the secretion of heparanase into the medium when added to HUVECs. (**B**) The ALKP level was evaluated in the above-described experiment. While dexacort increased, peptide 6 reduced the level of ALKP in the medium when added to HUVECs. All the assays were performed in triplicates. The results represent the median and range of three experiments.* *p* < 0.05, ** *p* < 0.005.

As endothelial cells are a vital part of the bone tissue, we evaluated the effects of dexacort and heparanase inhibitory peptide in HUVECs. As in osteoblasts, dexacort increased the heparanase level and reduced the ALKP levels in HUVECs. The effects were significantly attenuated by heparanase inhibitory peptide 6 (Figure 4A,B).

### 3.5. Dexacort Increases Levels of Coagulation Parameters and Decreases Markers of Osteoblasts Activity in Mice Bone Tissue Microcirculation

**Figure 5:**
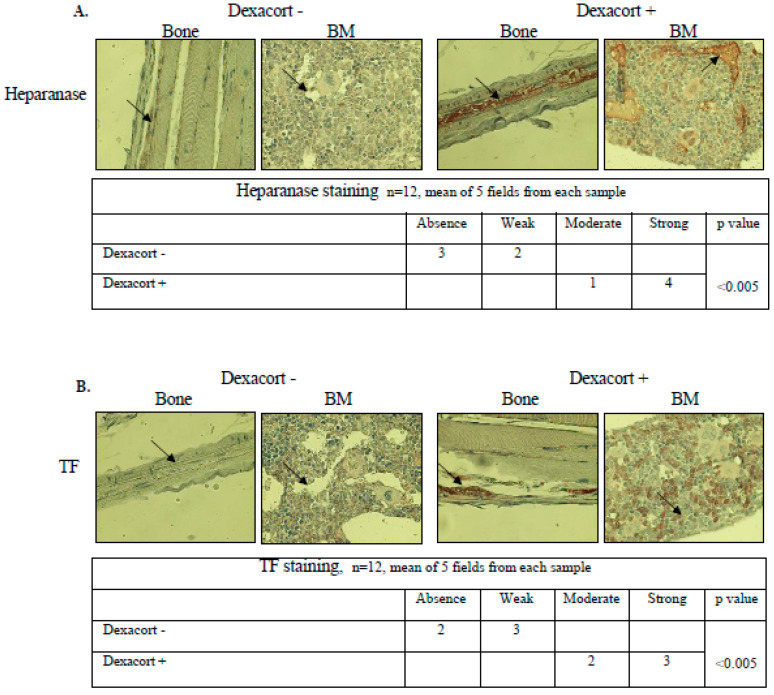

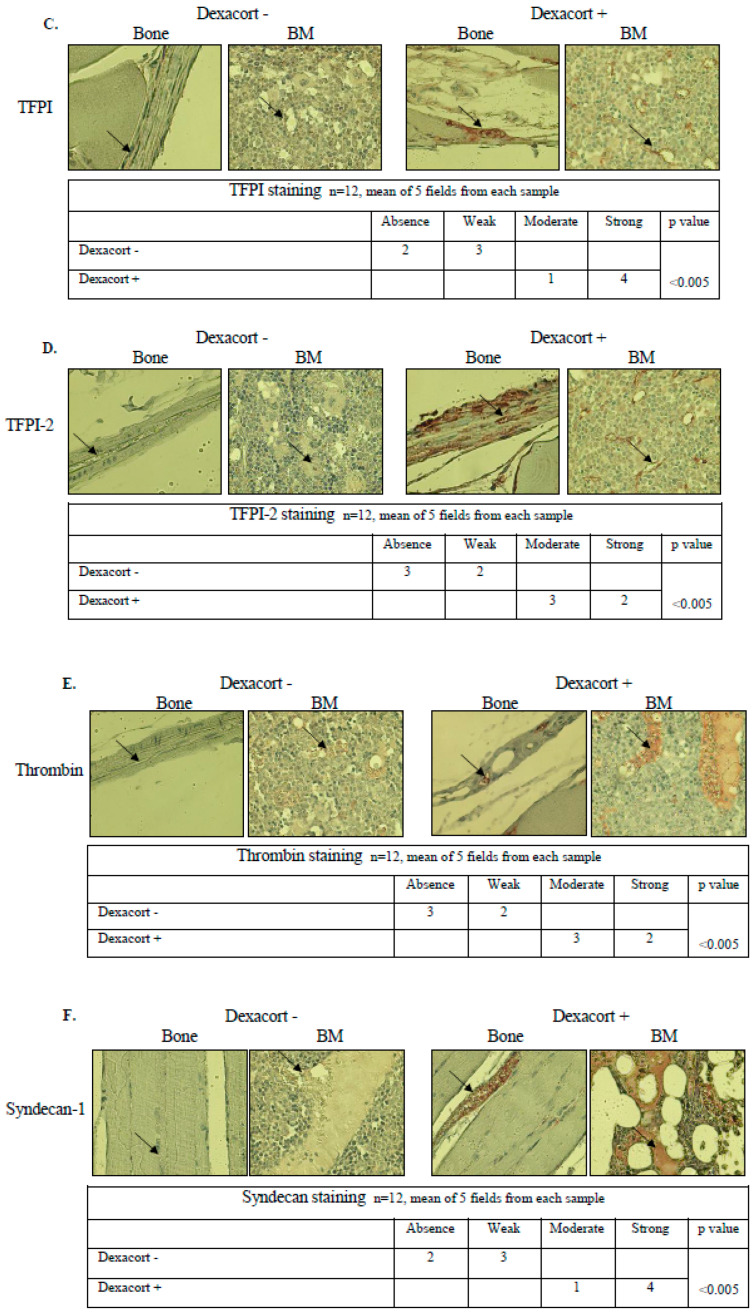

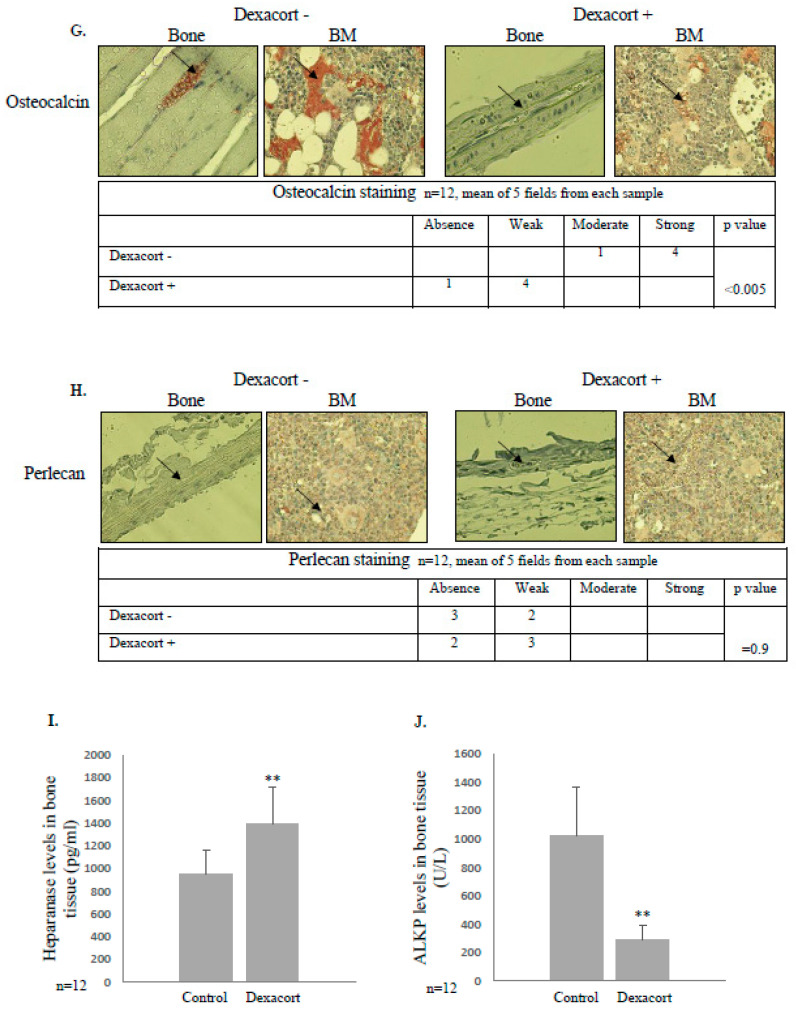
Dexacort increases the levels of coagulation parameters and decreases the markers of osteoblast activity in mice bone tissue microcirculation. (**A**) Immunohistochemical staining was performed in proximal bone tissue of mice. The control group (n = 6) received water without any drug for one month. The treatment group (n = 6) received water with the addition of dexacort (equivalent to a human therapeutic dose of 12 mg/day) for 1 month. The water was replaced every 48 h. At the end of the trial, the mice were sacrificed and the posterior proximal leg bones harvested. Slides were stained to the following five coagulation parameters: heparanase, TF, TFPI, TFPI-2, and thrombin, to two heparan sulfate proteoglycans that heparanase degrades; syndecan-1 and perlecan, in addition to osteocalcin, which is a marker of osteoblast activity. A significant increase in all the coagulation parameters, including syndecan-1, and a significant decrease in osteocalcin were observed in bone tissue of the treatment mice group as compared to the control mice group, mainly in the bone microcirculation and bone marrow sinusoids. The contiguous table shows the indicated protein staining intensity in the cells. Significance was determined by the Mann–Whitney U test. Representative images were visualized through ×50 magnitude, with a 0.82 MDC objective lens, captured with a Nikon E995 digital camera (Nikon, Tokyo, Japan), and processed with Adobe Photoshop software (Adobe Systems, San Jose, CA, USA) (**A**–**G**). No effect was found on the level of perlecan (**H**). In addition, bone samples were pulverized with a hammer in liquid nitrogen, extractions were centrifuged at 13,000× *g* for 15 min, and the supernatant was evaluated. The heparanase levels determined by ELISA were significantly higher and the ALKP levels were significantly lower in the study group as compared to the controls (**I**,**J**). All the assays were performed in triplicate. The results represent the median and range. The Mann–Whitney U test was used. ** *p* < 0.005.

In order to evaluate the impact of dexacort, which is known to induce osteoporosis, on the bone coagulation parameters and osteoblast activity, we used a mouse model as described below. The coagulation parameters included heparanase, TF, TFPI, TFPI-2, and thrombin. Osteocalcin is produced by osteoblasts and is often used as a marker of the bone formation process. Heparanase is known to degrade the heparan sulfate proteoglycans syndecan -1 and perlecan [29] and to up-regulate the syndecan–1 level [30]. Dexacort at a human therapeutic concentration of 6 µg/5 cc was added to the drinking water of six ICR mice (no specific genetic background), while six ICR mice without dexacort addition formed the control group. A 35 g mouse drinks approximately 5 cc water per day for an equivalent human dose of 12 mg/70 kg/day. The drinking water was replaced every 48 h and the experiment ended after 1 month. The levels of heparanase, TF, TFPI, TFPI-2, thrombin, and syndecan-1 were higher in the dexacort group, mainly in the microcirculation of the bone and bone marrow. In contrast, the levels of osteocalcin and ALKP were reduced in the bone tissue. No effect was found on the level of perlecan (Figure 5).

### 3.6. Malignancy, Steroids, and Inflammation Induce Coagulation Activation in Human Bone Microcirculation

**Figure 6:**
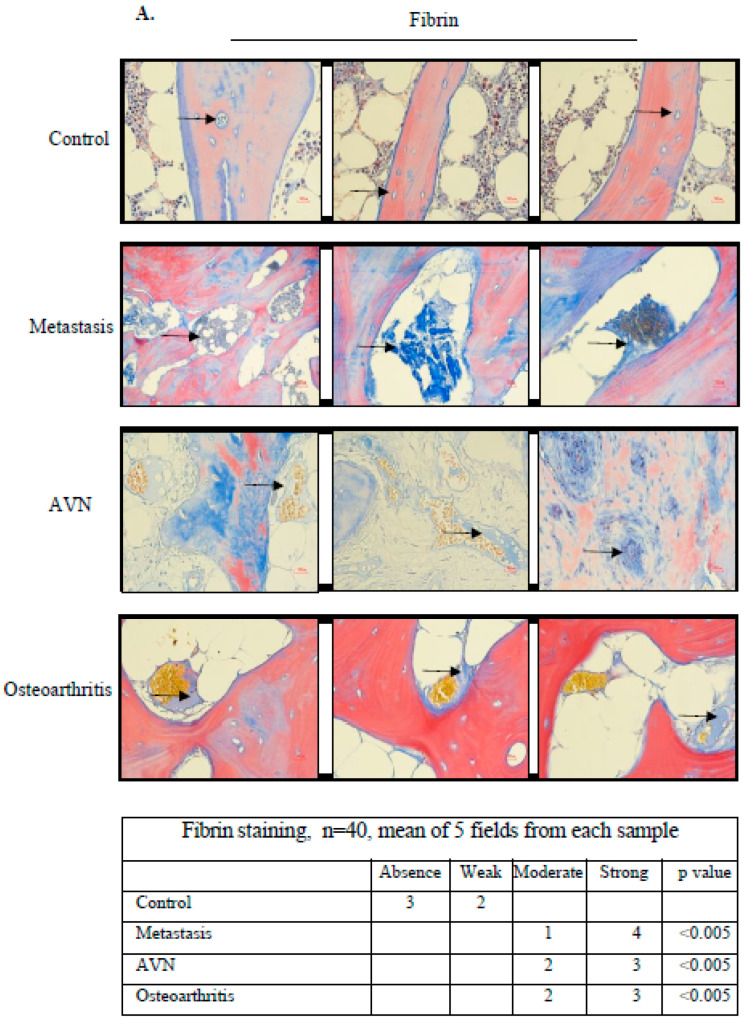

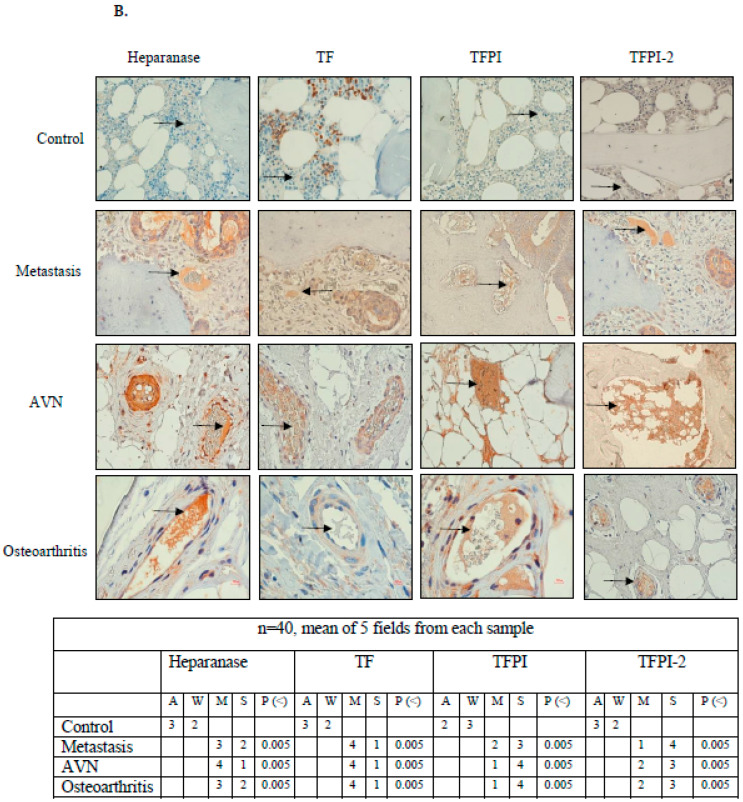
Malignancy, steroids and inflammation induce coagulation activation in human bone microcirculation. After obtaining written informed consent, the bone biopsies of 40 patients were studied: ten cases of bone metastasis of carcinoma origin, ten cases of avascular necrosis (AVN) of the femur head due to steroids, and ten cases of osteoarthritis of the femur head. These biopsies were taken at the beginning of femur surgery. Ten cases of diffuse large cell lymphoma without bone or bone marrow involvement, which formed the control group, were harvested during the bone marrow biopsy procedure during staging. There was no difference in gender, age, concomitant illnesses, or anti-aggregant use in the four groups. Data are presented in Table 1. The bone biopsies were studied using specific staining to fibrin with Martin Scarlet Blue (MSB) and immune-staining to heparanase, TF, TFPI, and TFPI-2. We found that the blood vessels in the study group were intensely dilated in the bone tissue and bone marrow sinuses ((**A**,**B**), indicated by black arrows). In MSB, staining fibrin is blue and erythrocytes are yellow. Fibrin was present at a significantly higher level in the study group microcirculation as compared to the control. As the staining of MSB is specific to fibrin and does not stain fibrinogen, the fibrin found in the microcirculation represents micro-thrombi (**A**). Increased levels of heparanase, TF, TFPI, and TFPI-2 in the study group, as compared to the control, were found. Increased levels of heparanase and TF may induce activation of the coagulation system (**B**). Contiguous tables show the indicated protein staining intensity in cells. Significance was determined by Mann-Whitney U test. Representative images were visualized through x10 magnitude, with a 0.82 MDC objective lens, captured with a Nikon E995 digital camera (Nikon, Tokyo, Japan), and processed with Adobe Photoshop software (Adobe Systems, San Jose, CA, USA).

After obtaining written informed consent, the bone biopsies of 40 patients were studied: ten cases of bone metastasis of carcinoma origin involving the femur head, ten cases of avascular necrosis (AVN) of the femur head due to steroids, and ten cases of osteoarthritis of the femur head. These biopsies were taken at the beginning of femur surgery. Ten cases of diffuse large cell lymphoma without bone or bone marrow involvement formed the control group and were harvested during the bone marrow biopsy procedure during staging. There was no difference in gender, age, concomitant illnesses, or anti-aggregant use among the four groups. The data are presented in Table 1. The bone biopsies were studied using specific staining to fibrin with Martin Scarlet Blue (MSB) and by immune-staining to heparanase, TF, TFPI, and TFPI-2. We found that the blood vessels in the study group were intensely dilated in the bone tissue and bone marrow sinuses. Fibrin was present at significantly higher levels in the study group as compared to the control. As the staining of MSB is specific to fibrin and does not stain fibrinogen, the fibrin found in the microcirculation represents micro-thrombi (Figure 6A). the increased levels of heparanase and TF found in the study group as compared to the control may induce the activation of the coagulation system (Figure 6B).

## 4. Discussion

In the present study, we evaluated the effects of steroids and malignant milieu on osteoblasts, HUVECs, mice bone, and human bone. We found that dexacort and malignant medium increased heparanase in a dose-dependent manner and reduced osteoblast activity, as indicated by a lower level of ALKP. The effect could be further enhanced by the interaction of heparanase with TF using peptide 16AC and dramatically attenuated when peptide 6 inhibited the interaction between the heparanase–TF complex. The effect of dexacort and attenuation by peptide 6 was also demonstrated in HUVECs. At this stage, we decided to evaluate the effect of dexacort in an animal model. Mice under the effect of a human therapeutic dose of dexacort (equivalent to 12 mg/day) for 1 month exhibited significant changes in their bone tissue. Interestingly, the main changes were found in the microcirculation endothelial cells and lumen. The levels of the coagulation factors heparanase, TF, TFPI, TFPI-2, and thrombin were increased while the markers of osteoblast activity, including osteocalcin and ALKP, were reduced. Syndecan-1 expression, which heparanase up-regulates and degrades, was also increased, while no effect was found on another heparan sulfate proteoglycan that heparanase degrades—perlecan.

In order to gain clinical evidence of the activation of the coagulation system in the microcirculation of the bone in malignancy, steroids treatment, and inflammation, we studied the bone biopsies of patients with bone metastasis, AVN induced by steroids, and osteoarthritis. We found in the microcirculation not only an increase in heparanase and TF that may activate the coagulation system but also micro-thrombi of fibrin residing in extensively dilated blood vessels.

We previously demonstrated that higher levels of the tetrad heparanase, TF, TFPI, and TFPI-2 are present in hypercoagulable states as malignancy, recurrent abortions, pregnancy, and infection [23,24], mainly in the tissues’ microcirculation. We also demonstrated that heparanase up-regulates the expression of TF [14], TFPI and itself using a positive feedback mechanism [26]. According to the present results, dexacort and malignant milieu induce, in the small blood vessels and sinuses of the bone marrow, a hypercoagulable phenotype that can potentially contribute to microcirculation thrombosis and bone tissue damage. In parallel, these conditions reduce the osteoblast activity that hampers normal bone remodeling. Microcirculation thrombosis may also contribute to patients’ bone pain. According to our results, intervention with anticoagulant drugs to prevent micro-thrombosis or using specific heparanase inhibitors should be further evaluated as a modality to reduce bone pain in these pathologies. Although unfractionated heparin (UFH), which inhibits heparanase, is known to induce osteoporosis, other lower-molecular-weight heparins that also strongly inhibit heparanase do not exert osteoporosis [31]. Thus, effect of UFH on bone is possibly related to its high molecular weight, enabling the interaction and replacement of bone ECM, and not due to the inhibitory effect on heparanase. We previously demonstrated that heparanase and TFPI are up-regulated following a mechanical AVN model in rats and the levels are normalized by treatment with bisphosphonates [32]. Hence, other treatment modalities to reduce heparanase may be evaluated concomitantly.

The induction of hemostatic system activation by malignancy was implicated by numerous mechanisms involving coagulation factor production by the liver and local endothelial cells [6]. The main mechanism of steroids’ induction of a hypercoagulable state was shown to be elevated levels of von Willebrand factor (vWF) and plasminogen activator inhibitor-1 (PAI-1) [33], both produced by endothelial cells. In the current study, we add another mechanism of activation of the coagulation system by steroids: heparanase, which mainly resides in platelets and neutrophils [34] and is also produced by endothelial cells that may exert a local prothrombotic phenotype.

Heparanase was previously demonstrated to affect bone formation. Transgenic mice that overexpress heparanase showed markedly increased trabecular bone mass, cortical thickness, and bone formation rate, implying that heparanase may support the process of injured bone tissue regeneration [35]. Po-yo et al. evaluated two different dosages of recombinant mouse heparanase and vehicle control that were delivered via an osmotic pump to provide a continuous topical infusion in a mouse bone defect model at the distal femoral metaphysis. Both the bone mineral density and cortical bone volume fraction showed the best healing outcome with low-dose heparanase, and an even worse outcome as compared to the control in high-dose heparanase, implying a biphasic effect of its mode of action [36]. Hence, low heparanase levels may enhance, while high levels may hamper, bone healing. In our study, we found that steroids and malignant milieu are in correlation with reduced osteoblast activity, implying that the heparanase level present in these two pathological conditions is too high, hampers bone healing, and supports therapeutic modalities to inhibit heparanase.

Filipowska et al. described the role of the bone vasculature in proper systemic functioning and pathology in a comprehensive review [37]. They elaborated on several mechanism affecting bone vessels, such as the low number of endothelial progenitor cells in AVN [38], effect of a low estrogen level in postmenopausal osteoporosis [39] and induction of bone marrow microangiopathy in diabetic mellitus [40]. In the current study we add the aspect of microcirculation thrombosis affecting bone pathology.

One of the limitations of this study is that we only evaluated the effect of dexacort. Other steroids should be evaluated regarding the effect on bone microcirculation thrombosis. Another limitation is that the malignant cell lines that were evaluated only included breast and lung cancers. Other malignant cell types should be further evaluated and compared.

## 5. Conclusions

**Figure 7:**
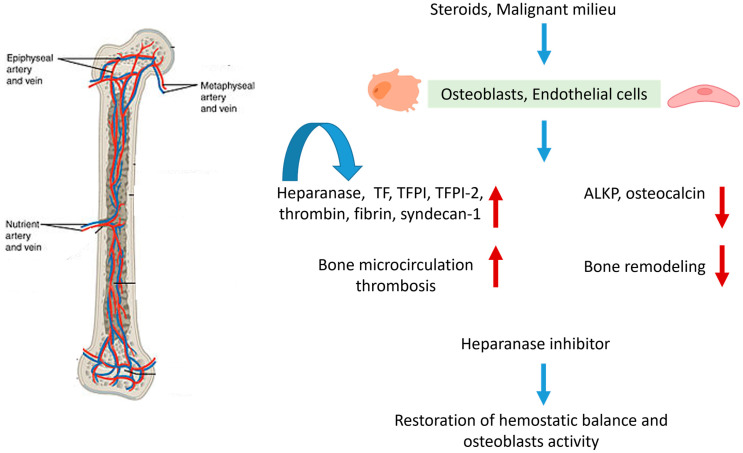
Schematic summarization. (**Left**) The blood supply by the nutrient vessels to the bone and bone marrow is the same, thus both can be considered one organ. (**Right**) Steroids and malignant milieu in osteoblasts and endothelial cells induce upregulation of proteins involved in microcirculation thrombosis and downregulation of proteins involved in bone remodeling. Heparanase up-regulates itself using a positive feedback mechanism. Inhibition of heparanase restores the hemostatic balance and osteoblast activity.

In the present study, we show for the first time the effects of steroids and malignant milieu on osteoblasts and endothelial cells regarding the upregulation of heparanase and downregulation of osteoblast activity (schematic summarization is presented in Figure 7). The effect can be enhanced by heparanase when interacting with TF, as demonstrated by the effect of peptide 16AC in a positive feedback manner, and attenuated by the inhibition of the interaction between heparanase and TF, as demonstrated by the effect of peptide 6. A mouse model treated with oral steroids and human bone biopsies strengthened our results and showed dilatation of the microcirculation blood vessels, local activation of the coagulation system, and micro-thrombi formation, which may have further hampered bone tissue recovery and enhanced bone pain. Modulating the bone local heparanase level may prove a strategy for improving bone tissue healing.

## Figures and Tables

**Table 1:** Demographic characteristics of study group.

	Control *n* = 10	Metastasis *n* = 10	AVN *n* = 10	Osteoarthritis *n* = 10	*p* Value
**Female gender**	5/10	4/10	5/10	6/10	0.7
**Age, years (median ± SD)**	66 (14)	68 (12)	67 (17)	66 (15)	0.5
**Concomitant diseases** Diabetic mellitus type 2 Ischemic heart disease Hypertension Malignancy Acetyl salicylic acid use Acetyl salicylic acid and clopidogrel use	5/10 4/10 4/10 NA 4/10 1/10	4/10 3/10 6/10 NA 4/10 0/10	6/10 4/10 5/10 0/10 5/10 2/10	6/10 3/10 4/10 0/10 4/10 1/10	0.7 0.7 0.8 0.6 0.7 0.8

NA: nonsignificant.

## Data Availability

Data can be provided upon reasonable request by contacting the authors.
